# Successful resection of hepatocellular cancer not amenable to Milan criteria and durable complete remission induced by systemic polichemotherapy after development of metastases – should we think about revising the current treatment guidelines in selected patients?

**DOI:** 10.1186/1477-7819-11-236

**Published:** 2013-09-22

**Authors:** Ivana Rados, Sasa Badzek, Hilda Golem, Juraj Prejac, Irma Gorsic, Domina Kekez, Niksa Librenjak, Stjepko Plestina

**Affiliations:** 1 University of Zagreb Faculty of Medicine, Salata 3, 10000, Zagreb, Croatia; 2 Department of Oncology, Division of Gastrointestinal Malignancies, University Hospital Center Zagreb, Kispaticeva 12, 10000, Zagreb, Croatia

## Abstract

**Objectives:**

To refresh clinical diagnostic and therapeutic dilemmas in patients presenting with hepatocellular cancer (HCC) and to report a rare success of systemic polichemotherapy in metastatic HCC.

**Methods:**

Case report of a patient with successfully resected HCC although initially deemed inoperable according to current guidelines, and who was successfully treated by systemic polichemotherapy after development of metastatic disease, resulting in a sustained complete remission.

**Results:**

We describe a 71-year-old female with HCC initially treated by atypical liver resection, although not amenable to initial surgery according to current treatment guidelines, which resulted in 6 months disease-free interval. After development of pulmonary metastases, the patient was treated by systemic polichemotherapy, due to local unavailability of novel biologic agents. After 3 months of chemotherapy biochemical remission was confirmed, and after 10 months of active treatment complete radiological remission was verified according to Response Evaluation Criteria in Solid Tumors (RECIST) criteria, now exceeding 9 months in duration.

**Conclusion:**

There is an increasing body of evidence that criteria for surgical interventions in HCC should be revised and expanded, and our case is an example of such an approach. Although novel biologic therapies are not widely available in all regions of the world due to their cost, currently there are no hard recommendations for use of chemotherapy in such areas. Since this is a large problem in clinical practice, we conclude that chemotherapy should be offered to selected patients of good performance status if novel agents are unavailable.

## Background

Cancer of the liver is one of most common malignancies worldwide [1]. It emerges in fields of carcinogenesis in cirrhotic liver due to any cause, and therefore is one of paradigms of viral causes of cancer [2-7]. Other risk factors implied include chronic alcohol consumption, metabolic liver diseases, cryptogenic cirrhosis, and aflatoxin B1 [8,9]. It can also occur sporadically in patients without any identifiable cause, in approximately 20% of cases.

Since its common presentation is in patients with severe liver dysfunction, it is one of the deadliest cancers as well as one of the cancers which are hardest to treat.

In patients with localized disease, main ‘curative’ treatment modalities are surgery, embolization, chemoembolization, radiofrequency ablation and orthotopic liver transplantation, with liver transplantation being most beneficial in terms of survival, although some controversy exists whether radical resection should be the treatment of choice [10,11]. Thirty to forty percent of patients with localized disease are deemed eligible for curative intention and are selected according to Milan criteria, although recently there are published data suggesting that these criteria should be expanded [12,13].

However, in patients with metastatic disease, treatment options remain limited. Historically, chemotherapy was the only hope for prolongation of life in these patients, and polichemotherapy has not been shown to be beneficial in terms of survivorship compared to doxorubicin alone, although better response rates have been reported [14]. One of the possible reasons is presentation of disease in patients with severe comorbidities. With the emergence of novel biologic agents, primarily sorafenib, response rates became higher and overall survival has practically doubled, but still remains disappointingly low [15-17].

Novel biologic agents are very expensive, and therefore are not yet available in all parts of the world. Recommendations of the European Society of Medical Oncology suggest use of chemotherapy in regions where no other options are available in medically fit patients [18].

We present a case of sporadic hepatocellular cancer (HCC), radically resected although not amenable to Milan criteria, and successfully treated with systemic chemotherapy after development of metastatic disease, producing durable complete remission.

## Case presentation

**Figure 1:**
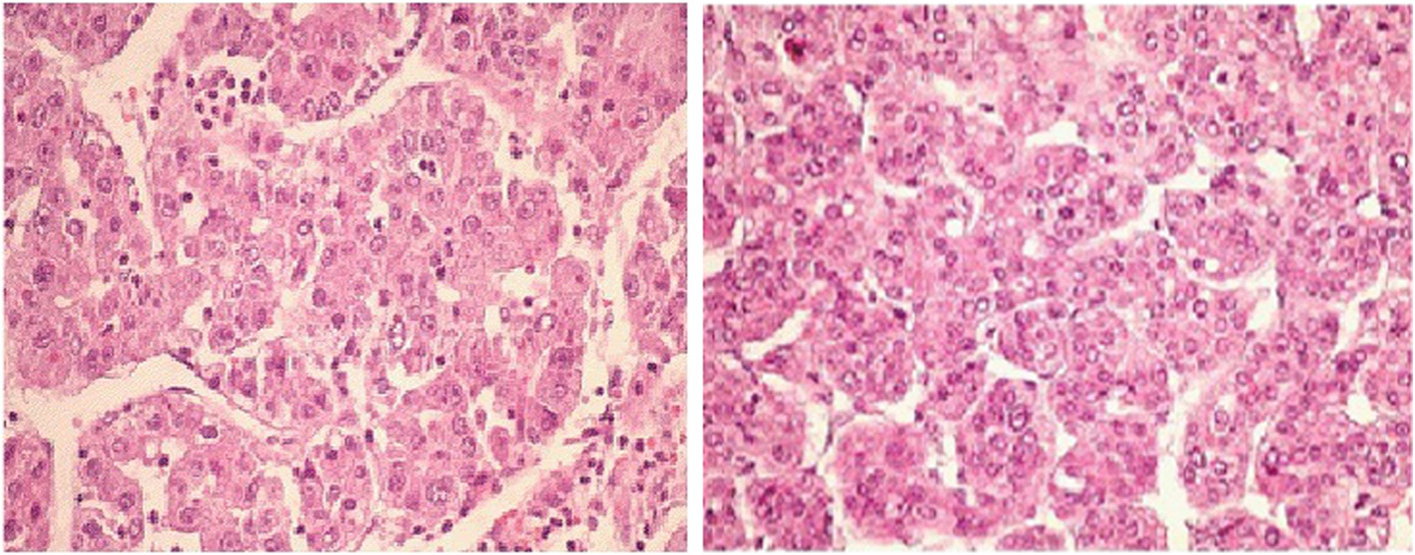
**Pathohistological review of hepatocellular cancer.** Clusters and trabeculae of atypical hepatocytes with hyperchromatic nuclei and angioinvasion are visible, surrounded by healthy liver tissue. Hematoxylin-eosin stain, magnification 400x, of bioptic pathohistological samples.

**Figure 2:**
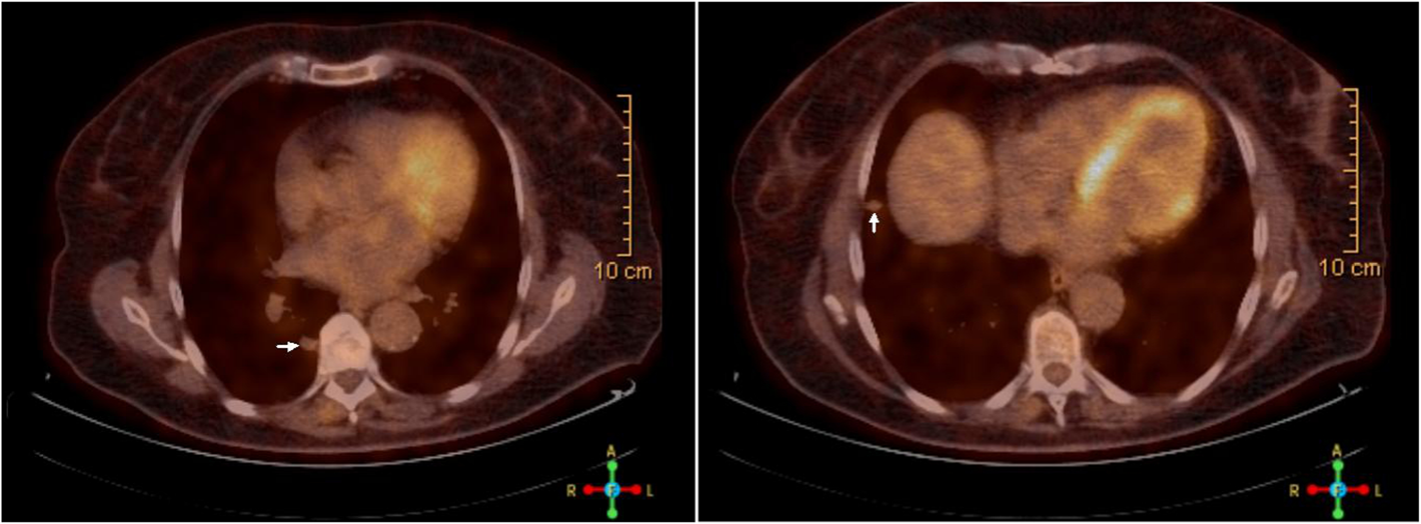
**Fluorodeoxyglucose-positron emission tomography scan of pulmonary metastases.** PET scan images reveal two larger metastases in the right lung: paravertebral (maximal Standardized Uptake Value = 1.9, 16 mm largest diameter) and peripheral subcostal (maximal Standardized Uptake Value = 2.9, 13 mm diameter), as indicated by white arrows. A few smaller nodules (fluorodeoxyglucose-positron emission tomography scan negative) in both lungs are also visible.

A 71-year-old female diabetic patient was diagnosed with incidental liver mass during liver ultrasound undertaken for other reasons in a small regional hospital. Initial alpha-fetoprotein (AFP) was >5000 ng/ml, and after adequate radiological evaluation (3-phase computed tomography scan) diagnosis of HCC was established, consisting of four liver nodules larger than 5 cm. No risk factors for HCC development were identified. Histological confirmation of the diagnosis was made, and the patient was referred to the experienced center (Figure 1). The tumor was classified as cT3aN0M0, IIIA stage. According to Milan criteria, the patient was not a candidate for liver transplantation. Since she was of excellent overall performance status and had no signs of chronic liver disease, atypical liver resection was attempted. According to the pathohistological review, surgical resection was adequate, ensuring >1 cm margin. Since no signs of the disease were found during postoperative re-evaluation, no further action was undertaken. After 6 months follow-up, and increase in AFP level was detected, and multiple pulmonary metastases were confirmed on consequent computed tomography and positron emission tomography scan (Figure 2). The patient was further treated with polichemotherapy according to the modified ECF regimen (epirubicin 50 mg/m^2^ on day 1, cisplatin 60 mg/m^2^ on day 1, and 5-fluorouracil 840 mg/m^2^ on days 1 to 5, given as a 21-day cycle). After three cycles of chemotherapy, biochemical remission was confirmed and radiological evaluation revealed a partial response. Chemotherapy was continued up to 11 cycles, when complete radiological remission was confirmed. An additional two cycles of chemotherapy were given, completing 1 year of treatment, to eradicate residual microscopic disease. Today, after 7 months of close follow-up, the patient is alive and still in complete remission (exceeding 9 months duration), although experiencing mild peripheral neurological toxicity.

## Conclusions

HCC is a deadly disease, with 5-year overall survival below 45% even when treated by liver transplantation in patients with pTNM stage IVA [19]. Whether current liver transplantation criteria should be expanded is a subject of an ongoing debate, as described earlier. On the other hand, successfully attempting surgical resection in a patient with locally advanced disease is much more challenging, and depends primarily on the surgeon’s experience. As we described in our patient, successful surgical resection was made after referral to an experienced center, although the patient was diagnosed and initially deemed inoperable in a small hospital. Another thing we would like to emphasize is that it is well established that radiological and biochemical criteria are sufficient for diagnosis of HCC. We could speculate that the relative inexperience of small centers without a consultant oncologist for diagnosis and treatment of HCC resulted in an unnecessary biopsy, which could be a possible cause of tumor dissemination. We would like to emphasize that a consultant oncologist should be involved in the diagnosis and treatment of patients with cancer from the very beginning, which could prove useful in the improvement of outcomes.

In patients with metastatic disease, treatment options are limited producing modest improvement in overall survival of about 3 months in a minority of patients, even when novel biologic agents are used. Single chemotherapeutic agents which have proven beneficial in terms of response rate higher than 10% are doxorubicin, 5-fluorouracil and cisplatin [20].

Polichemotherapy, with the PIAF (cisplatin, interferon-alpha, doxorubicin, 5-fluorouracil) regimen most commonly used, has no proven survival benefit over doxorubicin alone, although it doubles the response rates and can result in complete pathohistological responses in patients with initially inoperable HCC at a price of increased toxicity [21-23]. Since our patient was of excellent performance status and sorafenib was not available for treatment of HCC in our country because it is not covered by the national insurance policy, we decided to try a polichemotherapy regimen, which resulted in durable complete remission. Since no clear benefit of interferon therapy has been shown in patients with metastatic non-viral-related HCC, we treated the patient with a modified ECF regimen [24,25]. To the best of our knowledge, this is the first case of complete remission of metastatic HCC in a patient treated with systemic chemotherapy according to the ECF regimen. Only a few complete responses to tegafur and thalidomide-based chemotherapy have been described so far [26,27]. Pathological and radiological complete responses are well known to correlate with better progression-free survival and overall survival, which can be supported by our case as well.

Since it has been described that diabetic patients with HCC treated by metformin have a better prognosis, maybe this can be attributed to the response seen in our patient [28]. We could speculate that metformin could be active in HCC either through mTOR (mammalian target of rapamycin) inhibition, or by interfering with the energetic balance of tumor cells by suppression of oxidative phosphorylation via AMPK (**5’ adenosine monophosphate-activated protein kinase**), consequently enhancing the efficiency of chemotherapy in p53-deficient cells [29,30].

To conclude, surgical resection of borderline resectable tumors should be attempted in patients with excellent performance status in the hands of experienced surgeons, at least until transplantation criteria are revised. Maybe chemotherapy should not be left forgotten as a treatment modality in metastatic HCC, and should probably be offered to patients with excellent performance status in areas where sorafenib is unavailable. Chemotherapy efficiency in combination with metformin should probably be further tested in clinical trials.

## Consent

Written informed consent was obtained from the patient for publication of this Case report and any accompanying images. A copy of the written consent is available for review by the Editor-in-Chief of this journal.

## Abbreviations

AFP: Alpha-fetoprotein; ECF: Epirubicin, cisplatin, and 5-fluorouracil; HCC: Hepatocellular cancer.

## Competing interests

The authors declare that they have no competing interests.

## Authors’ contributions

IR drafted the manuscript and gathered the patient data. SB and HG drafted the manuscript, carried out diagnostic studies, and guided the patient therapy. JP, IG, DK, NL and SP were involved in drafting the manuscript. All authors read and approved the final manuscript.
